# Genome-Wide Identification, Evolution, and Expression Analysis of RING Finger Gene Family in *Solanum lycopersicum*

**DOI:** 10.3390/ijms20194864

**Published:** 2019-09-30

**Authors:** Liang Yang, Mingjun Miao, Hongjun Lyu, Xue Cao, Ju Li, Yuejian Li, Zhi Li, Wei Chang

**Affiliations:** 1 Vegetable Germplasm Innovation and Variety Improvement Key Laboratory of Sichuan Province, Horticulture Research Institute, Sichuan Academy of Agricultural Sciences, Chengdu 610066, China; yangliang0911@gmail.com (L.Y.); miaomingjun11@126.com (M.M.); dandelionlj@126.com (J.L.); yuejian_li@163.com (Y.L.); 2 Key Laboratory of Horticultural Crops Biology and Germplasm Enhancement in Southwest Regions, Ministry of Agriculture, Chengdu 610066, China; 3 Genome Analysis Laboratory of the Ministry of Agriculture, Agricultural Genomics Institute, Chinese Academy of Agricultural Sciences, Shenzhen 518124, China; hongjunlv2008@126.com; 4 Institute of Vegetables and Flowers, Shandong Academy of Agricultural Sciences, Shandong Province Key Laboratory for Biology of Greenhouse Vegetables, Shandong Branch of National Improvement Center for Vegetables, Jinan 250100, China; 5 College of Horticulture, Northwest A&F University, Yangling 712100, Shaanxi, China; xsunshine11@163.com

**Keywords:** *Solanum lycopersicum*, RING E3 ligases, phylogenetic analysis, collinearity, expression patterns, abiotic stress

## Abstract

RING domain proteins generally have E3 ubiquitin ligase activity and are involved in degrading their substrate proteins. The roles of these proteins in growth, development, and responses to different abiotic stresses have been described well in various plant species, but little is available on tomatoes. Here, we identified 474 RING domains in 469 potential proteins encoded in the tomato genome. These RING genes were found to be located in 12 chromosomes and could be divided into 51 and 11 groups according to the conserved motifs outside the RING domain and phylogenetic analysis, respectively. Segmental duplication could be the major driver in the expansion of the tomato RING gene family. Further comparative syntenic analysis suggested that there have been functional divergences of RING genes during plant evolution and most of the RING genes in various species are under negative selection. Expression profiles derived from a transcriptomic analysis showed that most tomato RING genes exhibited tissue-specific expression patterning. Further RT–qPCR validation showed that almost all genes were upregulated by salt treatment, which was consistent with the microarray results. This study provides the first comprehensive understanding of the RING gene family in the tomato genome. Our results pave the way for further investigation of the classification, evolution, and potential functions of the RING domain genes in tomato.

## 1. Introduction

Ubiquitination is a posttranslational protein modification that occurs in almost all eukaryotes. The target protein labeled with ubiquitin is degraded by the ubiquitin–proteasome system in a series of important cellular functions [1]. In plants, ubiquitin-mediated degradation is important in growth, signal transduction, responses to abiotic stress, embryogenesis, and senescence [2]. This process involves two successive steps: first, a polyubiquitin molecule chain is attached to the substrate protein, and second, the labeled protein is degraded by the 26S proteasome, a large multicatalytic protease complex. Ubiquitin (Ub) is a stable, highly conserved protein that consists of 76 amino acids and is expressed in almost all eukaryotes. The canonical process of ubiquitination starts with the activation of Ub by the E1 ubiquitin-activating enzyme, followed by the transfer of Ub to the E2 ubiquitin-conjugating enzyme to form a thioester-linked E2–Ub intermediate. The substrate-recruiting enzyme, E3 Ub ligase, interacts with E2–Ub and transfers the Ub to the target protein. The addition of Ub to the substrate protein is repeated by E3 ligase and allows the 26S proteasomal degradation system to recognize the substrate [3]. The Ub molecules are then released and recycled for the next round of ubiquitination [4]. A large number of E3 Ub ligases confer the specificity of ubiquitination [5]. For example, about 1600 genes (approximate 6% of the total genome) are involved in the Ub/26S proteasome system and related functions in *Arabidopsis* [6,7], including two members of the E1 Ub activating enzyme, 37 members of the E2 Ub conjugating enzyme, and over 1300 members of the E3 Ub ligase or components of E3 complexes. This large number of Ub-related proteins indicates that ubiquitination is the main regulatory pathway in many cellular processes.

The E3 Ub ligases can be divided into three groups based on their different catalytic domains: the HECT (Homology to E6-associated Carboxy-Terminus) group, the U-box group, and the RING (Really Interesting New Gene) group [4]. The RING domain is a specialized type of Zn-finger structure, consisting of 40–60 amino acids and characterized by eight spatially conserved cysteines (Cys) or histidine (His) metal-binding residues, which coordinate two zinc ions to form a cross-brace structure [8]. However, compared with proteins containing classical zinc-finger domains, which mediate interaction with DNA and RNA, the RING motif is only crucial in protein–protein interaction and in E3 ligases, allowing them to bind the E2 enzyme [9].

The RING domains can be mainly divided into two types based on the residue at metal–ligand position 5 (ml5): RING-H2 (His at the 5th position) and RING-HC (Cys at the 5th position). These are the two essential types of RING domain in plants. For example, the *Arabidopsis* genome contains 477 RING domains in 469 genes, including 241 RING-H2 type and 186 RING-HC types, and five modified RING domains: RING-v (25), RING-C2 (10), RING-D (10), RING-S/T (4), and RING-G (1) [10,11]. Most of these RING proteins can catalyze the formation of a polyubiquitin chain in the presence of the E2 conjugating enzyme in vitro [10]. In the rice genome, 488 potential RING finger protein genes have been identified by a bioinformatic analysis and divided into five groups: RING-H2 (281), RING-HC (119), RING-v (23), RING-C2 (2), and another 63 ungrouped genes [12]. The apple genome has 663 predicted proteins containing 688 RING domains, which can be divided into nine types, seven of which were first found in *Arabidopsis*, including RING-H2 (367), RING-HC (208), RING-C2 (10), RING-v (35), RING-D (1), RING-S/T (11), and RING-G (2), and two of which are specific to the apple genome, RING-mH2 (10) and RING-mHC (44) [13]. In the *Brassica rapa* genome, 715 genes encode predicted RING proteins that contain 731 RING domains and can be divided into eight types: RING-H2 (371), RING-HCa (215), RING-HCb (47), RING-v (44), RING-C2 (38), RING-D (10), RING-S/T (5), and RING-G (1) [14].

Recent studies have shown that RING-containing proteins play important roles in various biological processes in plants, including the tolerance of abiotic and biotic stresses, hormone signal transduction, growth, development, and so on. For example, NLA (Nitrogen Limitation Adaptation) protein is involved in sensing nitrogen limitation and the associated signaling pathway [15], SDIR1 (Salt-and Drought-Induced RING finger 1) ubiquitinates SDIRIP1 (SDIR1-interacting protein 1), which regulates the expression of ABI5 to affect seed germination and the salt stress response [16], AtAIRPs (*Arabidopsis* ABA-Insensitive RING Proteins), including AtAIRP1, -2, -3, and 4, are significantly induced by abscisic acid (ABA) and drought stress, and function as positive regulators of ABA-dependent drought tolerance [17,18,19,20]. In the absence of stress, KEG (keep on going) acts as a regulator of the ABA signaling pathway by degrading ABI5, whereas ABA promotes its self-ubiquitination and degradation to maintain the level of ABI5 [21,22,23]. ATLs (Arabidopsis Tóxicos en Levaduras) are a type of intronless genes (including *AtATL43*, *AtATL78*, and *AtATL80*) that encode a closely related RING finger proteins, and are involved in abiotic stress responses [24,25,26]. DRIP1 and -2 (DREB2A-interacting proteins 1 and 2) interact with and ubiquitinate DREB2A protein in the cell nucleus, implying that DRIP1 and -2 function negatively in response to drought stress in plants [27]. Another important E3 ligase is HOS1 (High Expression of Osmotically Responsive Gene 1), which mediates the ubiquitination of ICE1 and plays a key role in attenuating the cold stress response [28]. In tomato, SpRing is involved in salt stress and functions as a positive regulator of salt tolerance [29].

The domesticated tomato, *Solanum lycopersicum*, is both an economically important crop species and the model plant in studies of fruit development [30], domestication [31], and stress responses [32]. Although numerous RING gene family members have been identified and characterized in *Arabidopsis* and several other species, no comprehensive, systematic investigation of the RING-type protein family has been reported for any solanaceous crop. In the present study, we undertook a comprehensive analysis of the RING gene family in the tomato genome to explore its potential roles in organ development and responses to different abiotic stresses. Further analyses of the predicted gene structures, phylogenetic relationships, conserved motifs, chromosomal distributions, duplication events, evolutionary divergence, and expression patterns of these genes provide insight into their potential pivotal roles in diverse biological processes. Thus, our results lay the foundation for further research into the important biological functions of the RING domain proteins in tomato.

## 2. Results

### 2.1. Identification of RING Domain Proteins in S. lycopersicum

To identify as many RING finger proteins as possible in tomato, three strategies were used in this study. First, the 469 RING proteins reported in *Arabidopsis* were used as queries in the BLASTp program to construct a multiple-sequence alignment against the latest whole proteome of *S. lycopersicum*. Second, all the representative RING domains in *Arabidopsis* were transformed into the regular expressions and used as queries to search against the same tomato genome. Third, the RING-domain-related hidden Markov model (HMM) profiles were used to identify the RING domain family genes in the tomato genome. The retrieved nonredundant hypothetical protein sequences were submitted to the SMART website for domain identification, and then manually examined for the presence of the eight conserved metal–ligands. Based on this method, a total of 474 RING domains were identified in 469 predicted tomato proteins, including 464 proteins containing a single RING domain and five containing two RING domains (Additional File 1: Table S1). The length of the protein-coding regions in the identified tomato RING E3 ligase genes ranged from 180 bp for *Solyc09g074160* to 5664 bp for *Solyc09g055260*, and the number of amino acids ranged from 60 to 1888. The predicted molecular weights varied from 7.02 kDa to 211.13 kDa, and the isoelectric points of these proteins ranged from 3.96 to 10.4. The 474 predicted RING domains could be divided into seven RING types based on the amino acid residues at the eight metal–ligand positions and the distances between them: RING-H2 (248), RING-HCa (142), RING-HCb (21), RING-v (40), RING-C2 (20), RING-S/T (2), and RING-G (1). The RING-D type, which is encoded in the *Arabidopsis* genome, was not detected in the tomato genome. We also identified 18 proteins containing RING domains that were categorized as ‘incomplete’ RING domains because one or more metal–ligands was lacking or modified (Additional File 1: Table S2). Four RING domains belonging to the RING-H2 type (3) or RING-HCa type (1) were localized to chromosome 0 of the tomato genome and were not considered further (Additional File 1: Table S3).

Among the 474 RING domains, the largest is the RING-H2 type, with 248 domains (52.3%), followed by the RING-HC type, with 163 domains (34.4%). According to the spacing between metal–ligand residue 7 (ml7) and ml8, the RING-HC domain can be divided into two subgroups: the RING-HCa and RING-HCb, with 142 and 21 members, respectively (Table 1). In addition to the canonical RING domains, the modified RING types (RING-v, RING-C2, RING-S/T, and RING-G) represent only 13.3% of the total predicted RING domains identified. The RING-v domain has 40 (8.4%) representatives and is characterized by a Cys residue at the ml4 position and a His residue at the ml5 position, which is reversed in the RING-HC type at the same residues. The fourth RING type, RING-C2, has 20 (4.2%) representatives and is characterized by Cys residues at the ml4 and ml5 positions, rather than the His residues seen in RING-H2. In the tomato genome, the two RING-S/T-type proteins differ from RING-HC in having a serine (Ser, S) residue at ml2 or ml6 instead of a Cys residue. The one RING-G-type protein differs from RING-HC by having a glycine (Gly, G) residue at ml5 instead of a Cys residue.

### 2.2. Conserved Spacing and AMINO acids Between Metal–Ligand Residues in Tomato RING Domains

**Figure 1:**
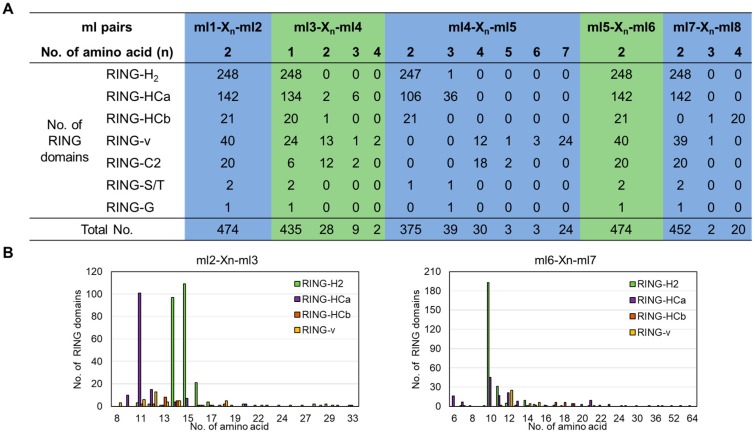
Spacing variations between metal–ligand (ml) pairs in S. lycopersicum RING domains. (**A**) The number of amino acids between each metal–ligand and the number of domains in which spacing variation occurs are shown. (**B**) Comparison of the numbers of amino acids in the loops between metal–ligands 2 and 3 and metal–ligands 6 and 7 of the RING-H2, -HCa, -HCb, and -v domains.

The representative RING domain is defined as an octet of metal-binding Cys and His residues that can chelate two zinc ions in a globular cross-brace structure, in which metal–ligand pairs 1 and 3 bind to one zinc ion and metal–ligand pairs 2 and 4 bind the other zinc ion [33]. This structure requires conserved spacing between the pairs ml1–ml2, ml3–ml4, ml4–ml5, ml5–ml6, and ml7–ml8, but variable spacing between pairs ml2–ml3 and ml6–ml7. To analyze the spacing patterns between the different metal–ligand pairs, we calculated the number of amino acid residues between the metal-binding sites (Figure 1A). All of the 474 putative tomato RING domains contained two amino acids (100%) between ml1–ml2 and ml5–ml6, 97.7% (463/474) contained 1–2 amino acid(s) between ml3–ml4, and 95.4% (452/474) contained two amino acids between ml7–ml8 excepted 21 RING-v type domains, which had 3–4 amino acids. Only 79.1% (375/474) of the RING domains had two residues between ml4–ml5, and the rest were RING-v type (spacing of 4–7 amino acids), RING-C2 type (spacing of 4–5 amino acids), and RING-HCa type (36 of them had three amino acids between ml4–ml5). In contrast, the number of amino acids between ml2–ml3 ranged from eight to 33 residues, and the most frequent number was 15 residues, whereas the number between ml6–ml7 ranged from six to 64, and the most frequent number was 10 (Figure 1B).

An analysis of the spacing variations revealed patterns within the same RING-type domains. For example, the RING-H2 domains had the highest frequencies of amino acids between ml2–ml3 (14 (97/248) or 15 (109/248)) and between ml6–ml7 (10 (193/248)). However, most of the RING-HCa domains had only 11 (101/142) amino acids between ml2–ml3 and six (16/142), 10 (45/142), 11 (17/142), or 12 (21/142) amino acids between ml6–ml7. Half (50%) the RING-v domains had seven amino acids between ml4–ml5, whereas 90% of the RING-C2 domains had four amino acids between these positions (Figure 1).

**Figure 2:**
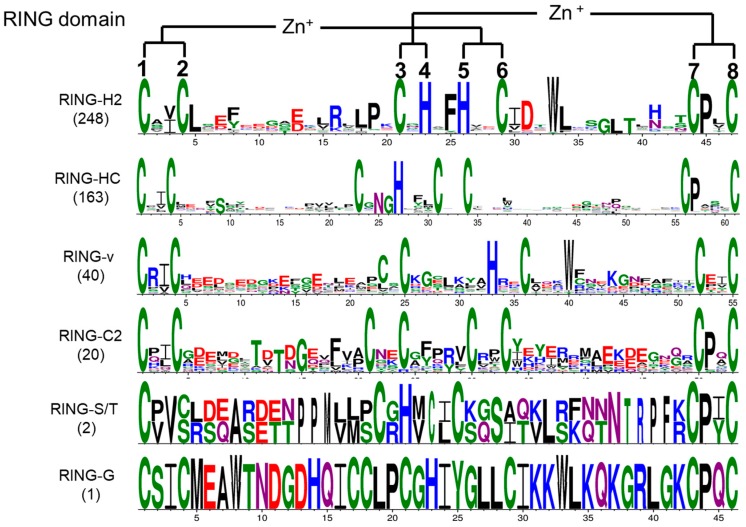
Schematic diagram of the conserved amino acids with metal–ligands in S. lycopersicum RING domains. Figures were created with the online WebLogo tool (http://weblogo.berkeley.edu/logo.cgi). Conserved metal–ligand positions and zinc-coordinating amino acid pairs are marked with asterisks.

To investigate whether any amino acids other than metal–ligand residues are conserved in the tomato RING domains, an alignment of all RING domains was analyzed, and sequence logos of the different representative RING domain types were constructed (Figure 2). The amino acid residue ahead of ml2 is most frequently Ile (I) or Val (V) among the different RING domain types. Another obvious conserved residue is Pro (P), which occurs at the second position after ml7 in over 95% of the RING domain types, except RING-v type, which has an aspartic acid (Asp, D) residue instead. In the RING-H2 domains, a phenylalanine (Phe, F) residue most frequently precedes ml5, a leucine (Leu, L) residue is always present next to ml2, and an Asp residue is usually present at the second position after ml6. An asparagine (Asn, N) followed by Gly is always found in front of ml4 in RING-HC. Over 96% of the RING-H2 and RING-v type domains have a tryptophan (Trp, W) residue at the fourth position after ml6. The amino acid after the ml1 position in the RING-v domain is almost always an arginine (Arg, R) residue.

### 2.3. Motif Distribution in Tomato RING Domain Proteins

To better understand the structural diversification of the tomato RING proteins, the full lengths of the 469 identified RING proteins were submitted to the SMART database to examine the conserved motifs outside the RING domains. As a result, 99 types of protein domains, other than the RING domain, were identified in all the tomato RING proteins and classified into 51 groups and their subgroups, based on their domain compositions and organization (Additional File 1: Table S4). Most of the groups had fewer members than group 1 (no additional protein domain, containing 157 members) and group 2 (a transmembrane domain with RING, containing 116 members), and over half the groups and subgroups had only 1–2 members. Among the conserved domains identified, some were considered to be protein–protein interaction domains, which may be related to substrate recognition, such as Ankyrin Repeats, BRCT, CRA, a coiled-coil domain, SPRY, TPR, Vwaint, and WD40. Others are considered to participate in the ubiquitination process, including CUE, GIDE, RWD, SINA, Ufd2P_corr, and ZNF_UBP. Numerous nucleic-acid-binding motifs associated with the RING domain were also identified, including CBFD_NFYB_HMF, DEXDc, HA2, HIRAN, KH, OB_NTP, PWI, ZnF-C2H2, RRM, WHIM1, ZnF-C3H1, and ZnF-NFX. In this analysis, some domains were predicted to function in binding metal–ions, such as Zn^2+^-binding domains (including Zinc_ribbon_9, ZnF_C2HC, ZnF_RBZ, SWIM, ZnF-CHY, Zinc_ribbon_6, and ZnF_NFX) and heavy-metal–ion-binding domains (including HMA). A number of domains linked to the RING motif are only found in the tomato or other plant genomes. For example, the RING domain with CBFD_NFYB_HMF is only found in the tomato, whereas the Cellulose_synt domain, HMA domain, JmjC domain, and WRC domain combined with the RING domain are specific in plant species, including *Arabidopsis thaliana*, *Brassica napus*, *Medicago truncatula*, *Oryza sativa*, *Zea mays*, etc., suggesting that they might perform the same functions in these plants.

### 2.4. Phylogenetic and Gene Structure Analyses of the Tomato RING Gene Family

**Figure 3:**
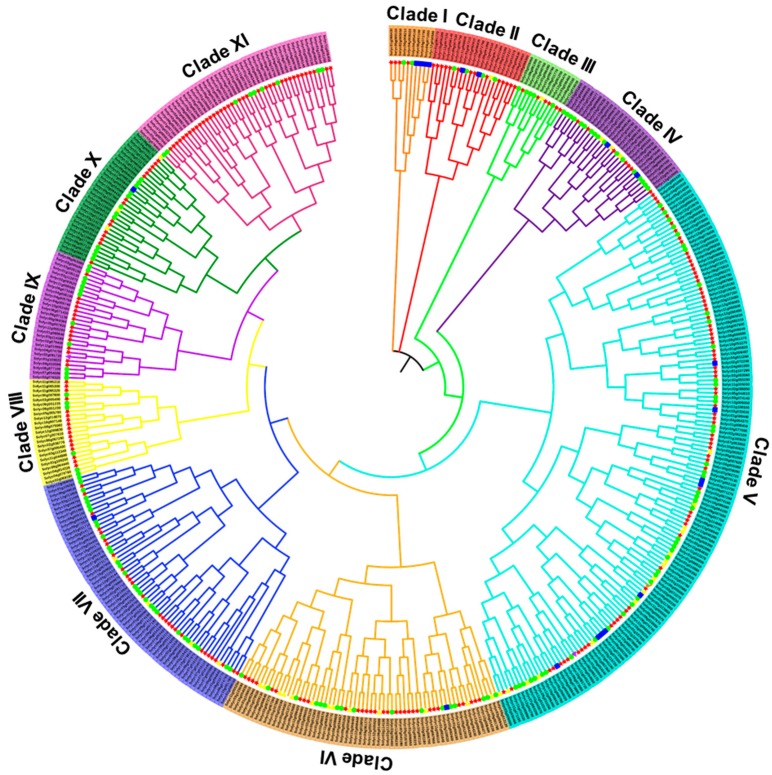
Phylogenetic analysis of *S. lycopersicum* RING domain genes. Phylogenetic tree was constructed with the NJ algorithm and 1000 bootstrap replicates. Differently colored arcs indicate different groups (or subgroups) of RING domain genes. Colored dots indicate different types of RING domain genes, including RING-H2 (red), RING-HC (green), RING-v (yellow), RING-C2 (blue), RING-S/T (purple), and RING-G (brown).

To study the evolutionary relationships of the RING gene family in tomato, a multiple sequence alignment of the 469 RING genes was used to construct a phylogenetic tree with the neighbor-joining (NJ) method (Figure 3). According to this phylogenetic analysis, the tomato RING proteins can be divided into 11 subgroups. Clade V had the most members, with 144 genes, whereas clade I had the least members, with only 10 genes. Although the RING domains can be classified into different groups according to the amino acid residues at the metal–ligand positions, we found no obvious phenomenon that clustered the same type of RING domain subfamilies, indicating that the sequences outside the conserved metal–ligand residues are very distinct across the different subfamilies.

An analysis of the exon/intron structures of the 469 tomato RING genes showed that most of the coding regions are separated by introns (Figure S1). However, 98 genes have no introns in their sequences. Interestingly, many of these occur closely on a single chromosome, indicating that they may be tandemly repeated sequences.

### 2.5. Chromosomal Localization and Gene Duplication Analysis of Tomato RING Domain Genes

**Figure 4:**
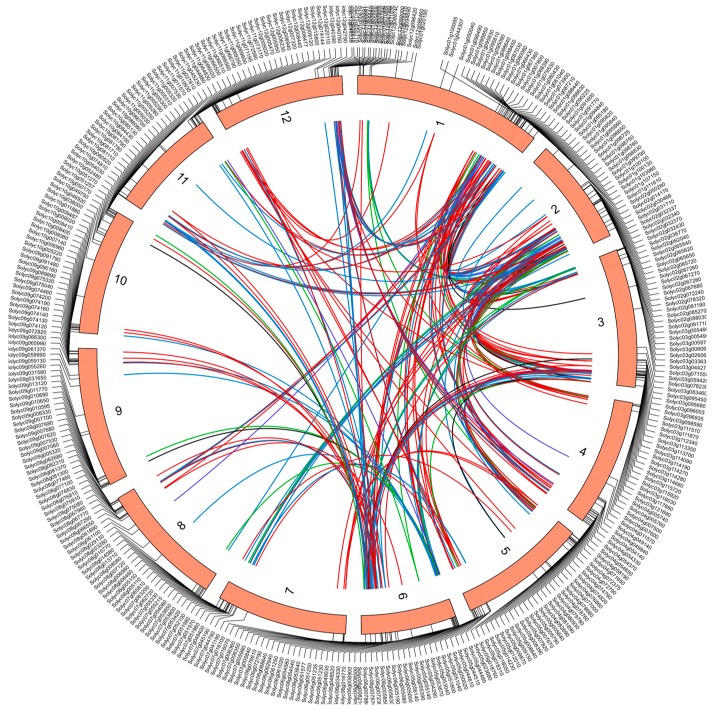
Schematic representations of the chromosomal distribution and interchromosomal relationships of *S. lycopersicum* RING domain genes. Chromosome numbers are indicated on the inner side of each chromosome. Gene names are indicated on the outside of each chromosome. Colored lines indicate duplications of different types of RING gene pairs, including RING-H2 (red), RING-HC (blue), RING-v (green), and RING-C2 (purple).

To analyze the localization of the RING domain genes in tomato, the coordinates of the 469 RING genes were extracted from the tomato genome annotation file. All the RING domain genes were distributed on the 12 tomato chromosomes, at different densities (Figure 4). Chromosome 1 contains the largest number of RING domain genes (64 genes), followed by chromosomes 2 and 3 (53 and 50 genes, respectively). Although chromosome 6 is the smallest chromosome (49.8 M) in the tomato genome, it contains the fourth-largest number of RING domain genes. The distribution of RING domain genes on the remaining chromosomes does not differ significantly, ranging from 27 to 38. Notably, most of the tomato RING domain genes are located at the ends of the chromosomes.

To analyze the gene duplication status, all the tomato RING domain genes were analyzed with the BLASTp and MCScanX software. Sixty tomato RING genes were grouped into 50 tandem duplication events. Ten tandem duplication events occurred on chromosome 1, suggesting that chromosome 1 contains a region in which the RING genes have been duplicated at high frequency. In total, 146 segmental duplication events involving 189 RING finger genes were also identified in the whole tomato genome (Additional File 2). These results indicate that the driving force behind the expansion for the tomato RING gene family has mainly been tandem and segmental duplication events. In order to further investigate the evolutionary selection pressure on the tomato RING gene family, the nonsynonymous (Ka) and synonymous substitutions (Ks) and the Ka/Ks ratios of RING gene pairs were calculated (Additional File 2). Only one pair of duplicated genes had Ka/Ks > 1, suggesting that the evolutionary selection pressures of RING genes in tomato tended to be purified.

**Figure 5:**
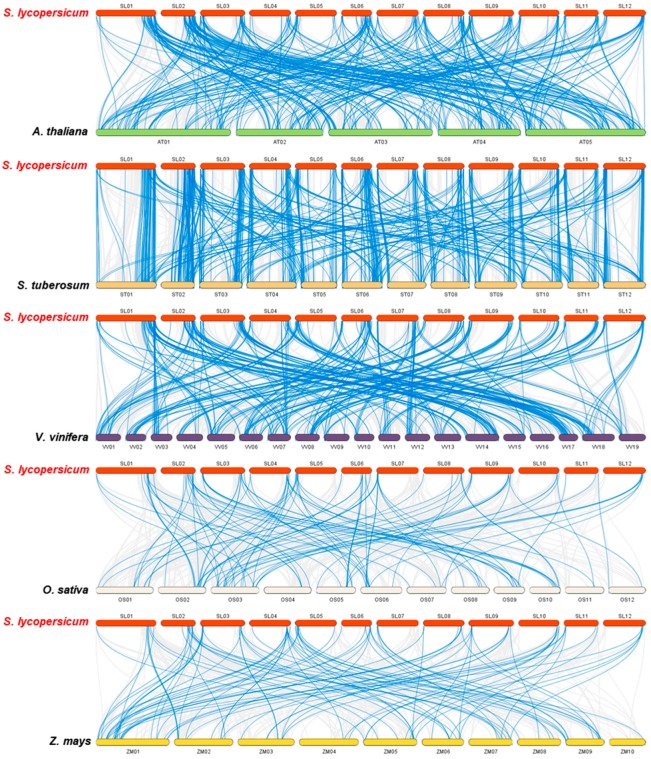
Synteny analysis of RING genes in tomato and five representative plant species. Gray lines in the background indicate the collinear blocks within the tomato and other plant genomes, and blue lines highlight the syntenic RING gene pairs. Plant species names with the prefixes “*S. lycopersicum*”, “*A. thaliana*”, “*S. tuberosum*”, “*V. vinifera*”, “*M. acuminate*”, “*O. sativa*”, and “*Z. mays*” indicate tomato, *Arabidopsis*, potato, grape, rice, and maize, respectively.

To further examine the phylogenetic mechanisms of the tomato RING family, we performed a comparative syntenic analysis of the tomato and five representative plant species, including three dicots (*Arabidopsis*, potato, and grape) and two monocots (rice and maize) (Figure 5). The dicot plants clearly shared more syntenic genes with the tomato (216 with *Arabidopsis*, 361 with potato, and 258 with grape) than the monocot plants (67 with rice and 61 with maize) (Additional File 3). The numbers of orthologous RING gene pairs between tomato and the other five species (*Arabidopsis*, potato, grape, rice, and maize) were 350, 552, 340, 100, and 97, respectively. Some of the tomato RING genes were found to have four or more syntenic gene pairs, including *Solyc03g114680*, *Solyc04g074820*, *Solyc08g081370*, and *Solyc11g010330*, indicating their important functions in the RING gene family during evolution. Furthermore, 27 syntenic gene pairs were identified between the tomato and all other species, suggesting that these orthologous RING gene pairs existed before their ancestral divergence and played pivotal roles in plants. One hundred thirty-one syntenic RING gene pairs were detected within the dicot species and 18 within the monocot species, suggesting that these gene pairs arose after the divergence of dicotyledonous and monocotyledonous plants. Further, most of the orthologous RING gene pairs had Ka/Ks < 1, suggesting that the tomato RING gene family has experienced strong purifying/negative selection pressure during its evolution (Additional File 3).

### 2.6. Cis-Elements in Promoters of Tomato RING Domain Genes

In order to further study the transcriptional regulation of tomato RING domain genes, we predicted the *cis*-acting elements in the 2-kb region upstream from the initiation codon (Additional File 4). The functions of predicted *cis*-elements can be mainly divided into three groups, including light responses (38 members), stress responses (21 members), and growth and development responses (12 members). Most promoters of tomato RING genes contained Box 4, G-box, and GT1-motif, which were involved in light responses. Furthermore, several *cis*-regulatory elements associated with stress responses such as ABRE, ARE, ERE, TGACG-motif, and WUN-motif, were detected in over 50% promoters of tomato RING genes. In addition, O2-site, circadian, and CAT-box were found in over 100 promoters of tomato RING genes, which were mainly involved in growth and development. These predicted *cis*-acting elements suggested that the tomato RING domain genes might function as important regulators in stress responses, as well as plant growth and development.

### 2.7. Expression Analysis of Tomato RING Domain Genes in Different Tissues and Fruit Developmental Stages

To investigate the differential expression of the RING domain genes in various tissues and their potential functions in tomato fruit development, we used a published tomato RNA-seq dataset from the Tomato Functional Genomics Database to determine the expression patterns of every tomato RING finger gene. A total of 444 tomato RING domain genes were identified in the transcriptomic data. However, the expression levels of 20 genes (including 13 RING-H2 types and seven RING-HC types) were not detected in all the tissues and were excluded from the analysis.

**Figure 6:**
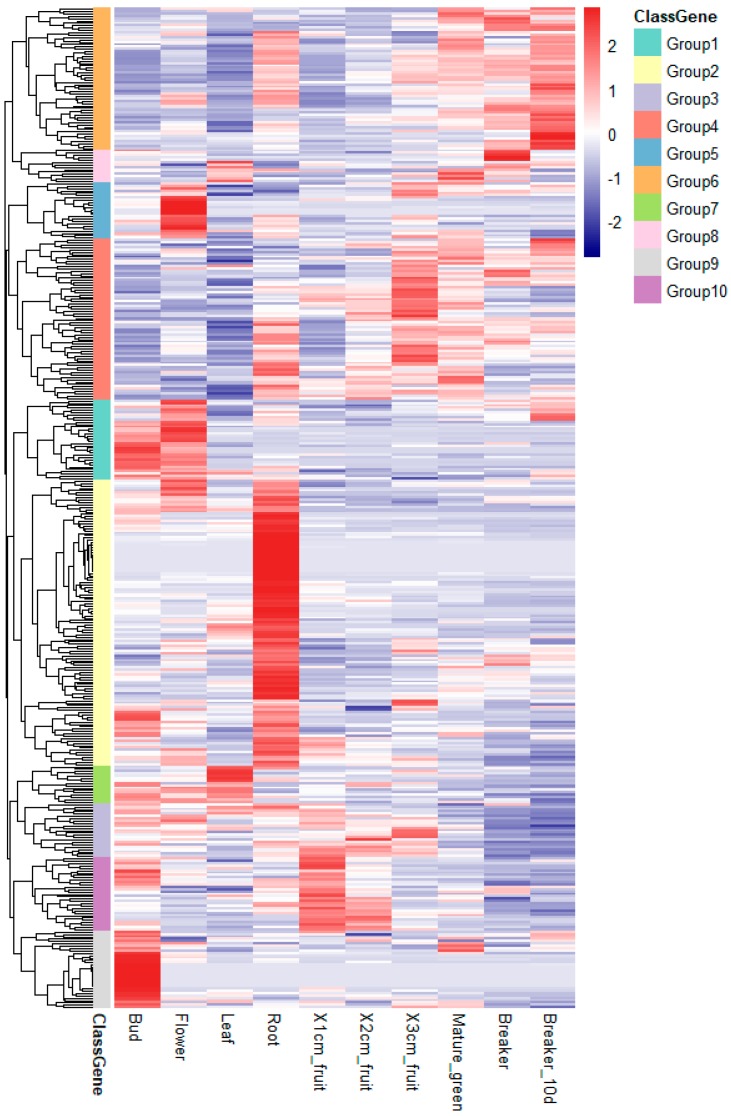
Expression profiles of 444 RING finger genes in different tissues and fruit developmental stages determined from RNA-seq data. FPKM-normalized values from RNA sequence data for different tissues of the tomato were used to construct the heat map. The RING finger genes are divided into 10 groups. The scale representing the relative signal values is shown beside the heatmap.

Based on the hierarchical clustering of their expression patterns, the remaining 424 RING finger genes were divided into 10 groups (Figure 6). Group 1 included 34 genes that were mainly expressed in bud or flower tissue. Group 2 contained 121 genes, and more than 80% of them were expressed >2-fold in root tissue. Group 5 included 24 genes, >60% of which were strongly expressed in flower tissue. Group 7 contained 16 genes and about half of them were strongly expressed in leaf tissue, in contrast to those expressed in flower tissue. Over 50% of the genes in group 9 (33 genes) were specifically expressed in bud tissue, at high levels. Groups 3 and 10 contained 23 and 31 genes, respectively, which were preferentially expressed in the early stages of fruit development (fruit 1–3 cm, mature green, breaker, and breaker after 10 days). Groups 4, 6, and 8 included 68, 60, and 14 genes, respectively, and more than 80% of these were expressed strongly in different fruit developmental stages, indicating that they may be directly or indirectly involved in fruit development in tomato.

**Figure 7:**
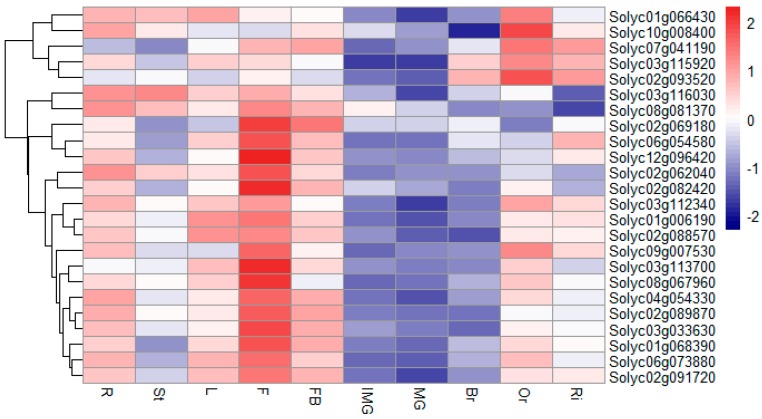
Expression profiles of 24 RING domain genes in various tissues and fruit developmental stages determined with RT–qPCR. Expression data were normalized to those of *SlACTIN*, log2 transformed, and then normalized by row. The scale representing the relative signal values is shown beside the heatmap. Abbreviations used for the tomato tissues: R, root; St, stem; L, leaf; F, flower; FB, flower bud; IMG, immature green fruit; MG, mature green fruit; Br, break fruit; Or, orange fruit; Ri, ripe fruit.

To further investigate whether the tomato RING type E3 ligases were involved in the different developmental stages, we chose 24 members of the RING finger gene family that were strongly expressed under salt stress in a microarray dataset (Additional File 6): nine RING-H2s, nine RING-HCs, three RING-vs, two RING-C2s, and one RING-S/T. We analyzed their transcription profiles in various organs and different fruit developmental stages with reverse transcription–qualitative PCR (RT–qPCR). As shown in Figure 7, most of the genes were preferentially expressed in vegetative tissues rather than in the fruit, except *Solyc02g093520*. Interestingly, over 60% of the RING finger genes were most strongly expressed in the flower. Furthermore, the detected tomato RING domain genes were more often expressed in the ‘orange’ stage rather than in the other stages of fruit development, indicating that more ubiquitination events occurred during this stage.

### 2.8. Expression of the Tomato RING Domain Genes in Response to Different Abiotic Stresses

**Figure 8:**
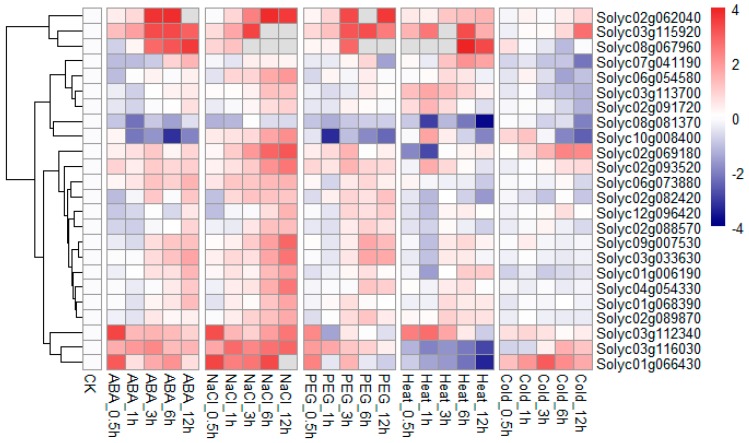
Expression profiles of 24 RING domain genes under various abiotic stresses determined with RT–qPCR. Tomato seedlings were treated with 200 μM ABA, 300 mM NaCl, 20% PEG, 40 °C heat, or 4 °C cold stresses for the indicated time. Expression data were normalized to those of *SlACTIN* and *Slβ-tubulin*, then get log2 transformed. The scale representing the relative signal values is shown beside the heatmap. CK, control sample.

Because plant E3 ubiquitin ligases are usually involved in plants’ responses to different abiotic stresses, we used RT–qPCR to examine the expression profiles of selected RING finger genes in tomato treated with ABA, salt, drought, heat, or cold stress, respectively. As can be seen in Figure 8, the expression levels of almost all the genes were upregulated by salt treatments, which were consistent with the microarray results. Of these genes, the expression of *Solyc03g112340* and *Solyc01g066430* was significantly increased within 0.5 h, whereas the transcripts of *Solyc03g115920*, *Solyc08g067960*, and *Solyc02g062040* were 183-, 57-, and 12-fold higher than the control values after salt treatment for 12 h.

The expression levels of most RING genes were upregulated after treatment with ABA or simulated drought for 3 h, as after the salt treatment, indicating that these genes may be involved in the same mechanisms in response to ABA-, drought-, and salt-treated stresses. On the contrary, the expression levels of *Solyc08g081370* and *Solyc10g008400* were decreased by nearly 80% after treatment with ABA or simulated drought for 1 h.

The transcript levels of more than half the genes analyzed were affected by heat stress: the upregulated genes included *Solyc02g062040*, *Solyc03g115920*, *Solyc08g067960*, *Solyc07g041190*, *Solyc03g113700*, *Solyc02g091720*, *Solyc10g008400*, *Solyc02g093520*, and *Solyc03g112340*, the downregulated genes included *Solyc08g081370*, *Solyc02g069180*, *Solyc02g082420*, *Solyc01g006190*, *Solyc03g116030*, and *Solyc01g066430*. Among the upregulated genes, *Solyc08g067960* showed a 36-fold increase over the control, with more highly expressed than other genes.

Following cold stress treatment, the transcript levels of *Solyc02g069180*, *Solyc03g115920*, and *Solyc01g066430* were nearly 5-, 6-, and 8-fold higher than those in the control, respectively. However, the transcript levels of *Solyc07g041190*, *Solyc08g081370*, and *Solyc10g008400* declined markedly after the cold stress treatment. No significant changes were observed in the expression of the other genes in response to cold stress treatment.

## 3. Discussion

As one of the most important gene superfamilies, RING finger genes are widespread in eukaryotes and have been substantially characterized in a diverse range of plant species, including 469 in *Arabidopsis*, 378 in rice, 399 in poplar, 65 in a green alga, and 715 in turnip [10,12,13,14,34,35]. Even after the complete genomic sequence of the tomato was published [36], its RING finger genes remained unidentified and uncharacterized. To improve our knowledge of the RING finger gene family in tomato, a genome-wide investigation was performed that identified 469 RING finger family members in the tomato genome database. The number was similar to those identified in *Arabidopsis* and provided potential candidates for further functional analysis.

In tomato, the RING finger genes comprise about 1.3% of the predicted protein-coding genes, which is similar with the proportions in rice (about 1.2%) and turnip (about 1.5%), but lower than that in *Arabidopsis* (about 2%). Therefore, genome duplication events might have contributed to the expansion of the RING gene family in *Arabidopsis* genome. The 469 RING domains can be subgrouped into seven different RING types, according to their amino acid compositions and the spaces between the eight metal–ligand residues. Although the numbers of RING-H2 and RING-HC domains are similar to the numbers in *Arabidopsis*, the tomato contains fewer modified RING domains, such as RING-v, RING-C2, and RING-S/T, than *Arabidopsis*, indicating that these RING domains have undergone specific alterations in the tomato genome or have been lost during evolution. For example, *Arabidopsis* has twice the number of RING-S/T type genes than that in the tomato genome. Besides, no RING-D domain was detected in the tomato genome, which has been reported as specific to *Arabidopsis* (Table 1).

According to the analysis of additional domains, the tomato RING finger proteins can be divided into 51 groups with their subgroups, which is almost twice the number in *Arabidopsis* (Additional File 1: Table S4) [10]. Most of these additional domains are predicted to function in protein-binding, ubiquitin- or nucleic-acid-binding, and metal–ion-binding, such as CUE, SINA, DEXDc, HA2, ZnF_C2HC, ZnF_RBZ, and ZnF-CHY. These diverse functional domains associated with RING domains may endow the RING E3 ligases with more variable roles, involving plant development and their responses to environmental stimuli. Among these additional RING-domain-associated domains, some patterns are specific to plants, such as Cellulose_synt, GYF, and WRC, suggesting that their biological functions are confined to plants. Moreover, some domains associated with the RING domains are only found in tomato. For example, although the CBFD_NFYB_HMF domain is a motif widespread among different species [37], the combination of this domain with the RING domain may be limited to tomato.

Plant genomes usually have a higher proportion of duplicated genes than other eukaryotes [38]. Tandem, segmental, and whole-genome duplication events are the primary causes of these expansions [39]. In this study, we found that over 50% of RING finger genes (249/469) are clustered as duplicated genes in the tomato genome (Figure 4), generating 50 tandem duplication events and 149 segmental or whole-genome duplication events. This suggests that segmental duplication rather than tandem duplication has played the predominant role in the expansion of the tomato RING finger gene family. Moreover, the Ka/Ks ratio is often used to interpret the direction and magnitude of the natural selection acting on various protein-coding genes [40]. In the tomato genome, only one pair of duplicated genes had Ka/Ks >1, suggesting that most of the tomato RING finger genes have experienced purifying selection pressure during evolution. A comparative genomic investigation showed that the tomato genome has experienced two whole-genome triplication (WGT) events, including an ancient triplication shared by the core eudicots and a recent event affecting the Solanaceae lineage, which caused the great expansion and strong evolution of speciation-related gene families [36]. The grape genome is known to have experienced only one triple genome duplication. In the grape genome, we found 224 orthologous RING finger genes, indicating that there should be >700 RING finger genes produced during the recent WGT event in tomato. However, only 469 tomato RING genes were identified, indicating that more than 30% of the duplicated RING finger genes were lost after WGT. Exon/intron structural gene variants are usually caused by insertion/deletion events and are useful for evaluating the evolutionary patterns of different gene families [41]. Introns are considered to be under weak selective pressure. Here, about 21% of the tomato RING finger genes had no introns, suggesting that these genes have evolved at a rapid rate.

The analysis of RNA-seq data showed that about 90.4% (424/469) of the RING finger genes displayed different expression levels in the various tissues and different fruit development stages (Figure 6). This suggests that the RING finger gene family contains functional variants that are involved in all stages of tomato growth and development. Our RT–qPCR results support this assumption. For example, the transcript levels of *Solyc02g062040* were high in the root, stem, leaf, and flower, but low in the different fruit growth stages. *Solyc093520* had an almost completely opposite expression pattern: low in tissues but high in fruit (Figure 7). Moreover, the RING E3 ligases in plants are reported to be strongly involved in the stress response pathways. One of the first indications of this important function was that the transcripts of the RING finger genes were upregulated in plants after treatment with abiotic stressors [16,19,20,42,43,44,45,46]. Among the 1500 E3 ligase genes in *Arabidopsis*, over 700 and 600 members are up- and downregulated in response to different abiotic stressors, respectively [6]. Our RT–qPCR results indicated that the expression of 24 RING genes, including nine RING-H2 types, nine RING-HC types, three RING-v types, two RING-C2 types, and one RING-S/T type, was markedly altered (up- or downregulated) in response to at least three types of abiotic stress. Furthermore, the presence of stress-responsive *cis*-acting elements in all types of tomato RING finger genes indicates that they function extensively as important regulators of abiotic stress responses and environmental adaptation (Additional File 4).

In conclusion, we comparatively analyzed the tomato RING finger gene family in this study. A total of 469 RING finger genes were characterized and classified into different groups or subgroups according to the RING domain type, additional domains, phylogeny, and their expression patterns. Chromosomal localization and synteny studies with different plant species will provide valuable information about the evolutionary features of the tomato RING finger gene family. Moreover, our classification of *cis*-acting elements and the analysis of gene expression will be useful for further determine the biological function of the RING finger genes, and to better comprehend their possible roles in mediating abiotic stress responses. This study provides a solid reference for the comparative analysis of the RING finger gene family in *Solanum* species and the selection of candidate genes for further functional analyses and genome editing in *Solanum* crops.

## 4. Materials and Methods

### 4.1. Identification of RING Finger Proteins in S. lycopersicum

To identify all the RING finger proteins in the tomato genome, the 469 RING proteins reported in *Arabidopsis* were used as queries in the BLASTp program against the latest *S. lycopersicum* whole proteome file in ITAG Release 3.2 from the Sol Genomics Network (SGN, https://solgenomics.net/) [47]. HMM profiles of related RING domain sequences (PF00097 for zf-C3HC4, PF12906 for RINGv, PF13639 for zf-RING_2, PF13923 for zf-C3HC4_2, PF13920 for zf-C3HC4_3, and PF15227 for zf-C3HC4_4) were downloaded from the Pfam database (http://pfam.xfam.org/) to identify the RING domain genes using the HMMER software (version 3.0) with the default parameters [48,49]. At the same time, regular expressions, which were designed to represent the different types of RING domains based on previous studies [10,14], were used to search for RING domain protein sequence in the tomato genome. All the redundant sequences were removed, and the remaining sequences were analyzed to confirm the presence of RING domains by submitting them to the SMART database (http://smart.embl-heidelberg.de/) and Pfam database with Perl script [50]. Each sequence was then inspected manually.

In the search results, some RING-related domains, such as PHD and LIM, were excluded because their ubiquitin ligase activities have not been confirmed. Those sequences predicted as RING domain sequences by SMART but lacking two or more metal–ligands were classified as incomplete RING domain proteins.

### 4.2. Phylogenetic Tree Construction and Structural Analysis of the RING Finger Genes

To study the phylogenetic relationship of the tomato RING finger genes, a multi-sequence alignment was constructed with the MEGA 7 software [51]. A phylogenetic tree based on the alignment was constructed with the NJ method with 1000 bootstrap replicates. The exon/intron structures of the tomato RING genes were determined with the online program Gene Structure Display Server (http://gsds.cbi.pku.edu.cn/) [52].

### 4.3. Chromosomal Localization, Gene Duplication, and Microsynteny Analysis of Tomato RING Finger Genes

The chromosomal localization data of each identified RING finger protein gene were retrieved from the GFF3 file in ITAG Release 3.2. The MCScanX software was used to identify duplicated and syntenic RING finger genes in the tomato genome, with the default settings [53]. The chromosomal distributions and microsyntenic relationships of the RING finger genes were visualized with the Circos software (version 0.69) [54].

To display the syntenic relationship of the orthologous RING finger genes in tomato and other selected species, syntenic analysis maps were constructed with the TBtools software (version 0.6668) [55]. The nonsynonymous and synonymous substitution rates (Ka and Ks, respectively) and Ka/Ks values of syntenic RING gene pairs were calculated with the ParaAT software (version 2.0) and KaKs_Calculator software (version 2.0) [56,57].

### 4.4. Cis-Regulatory Elements Prediction for Tomato RING Gene Promoters

The promoter sequence (2 kb upstream of the 5′UTR) of each RING domain genes was extracted from the tomato genome and submitted to the PlantCARE website (http://bioinformatics.psb.ugent.be/webtools/plantcare/html/) for *cis*-regulatory elements prediction [58]. The predicted *cis*-regulatory elements were classified according to their regulatory functions [59].

### 4.5. Expression Analysis of RING Finger Genes in Different Tissues and Fruit Developmental Stages of Tomato

The RNA-seq data for RING domain gene expression in four tissues (bud, flower, leaf, and root) and six fruit developmental stages (1 cm fruit, 2 cm fruit, 3 cm fruit, mature green, breaker, and breaker after 10 days) were retrieved from the Tomato Functional Genomics Database (http://ted.bti.cornell.edu/). The expression profiles, as fragments per kilobase per million reads (RPKM), of the tomato RING finger genes were extracted with Python script, clustered, and drawn with the pheatmap package in the R software (version 3.4), with Euclidean distances and the complete linkage method of hierarchical clustering.

### 4.6. Plant Materials and Treatments

The tomato (*S. lycopersicum* L. cv. MoneyMaker) plants were cultured in a greenhouse or growth chambers. Three-month-old tomato plants were used to analyze the transcript levels of the RING finger genes in different tissues. The roots, stems, leaves, flowers, and different fruit growth stages were collected for RNA extraction.

To analyze the transcript levels of the RING finger genes after different abiotic stress treatments, 15-day-old tomato seedlings were cultured in Murashige and Skoog (MS) liquid medium containing 200 μmol L^−1^ ABA, 300 mmol L^−1^ NaCl, and 20% (mass fraction) polyethylene glycol (PEG). The seedling plants were incubated at 4 °C and 40 °C to induce cold or heat stress, respectively. In all five treatment groups, the whole plants were collected after treatment for 0, 1, 3, 6, or 12 h. All the collected samples were frozen in liquid nitrogen and stored at −80 °C before cDNA synthesis and quantitative expression analysis.

### 4.7. RNA Isolation and RT–qPCR

Total RNA was extracted with the Quick RNA Isolation Kit (Huayueyang, Beijing, China), according to the manufacturer’s protocol. First-strand cDNA was synthesized from 1 μg of total RNA with the FastQuant RT Kit (Tiangen, Beijing, China), according to the manufacturer’s protocol. Real-time PCR was performed as described previously [46]. Briefly, the PCR amplification program consisted of an initial step at 95 °C for 3 min, followed by 40 cycles of 95 °C for 15 s, 60 °C for 10 s, and 72 °C for 30 s. The data were obtained with the CFX Manager software (version 3.6) (Bio-Rad, Hercules, CA, USA), and then normalized to the *SlACTIN* and *Slβ-tubulin* mRNA levels. All RT–qPCR experiments included two technical replicates and three independent biological repetitions. The relative gene expression values were calculated using the 2^−ΔΔCt^ method. Gene expression values were log2 transformed, and heatmaps were generated using the pheatmap package in the R software (version 3.4). The gene-specific primers were designed, according to the CDSs of genes, using Primer3 (version 4.1.0) [60]. The primers used in the RT–qPCR analyses are listed in Additional File 5.

### 4.8. Microarray Data Analysis

Microarray data for salt treatment (dataset NO. E051) was downloaded from the Tomato Functional Genomics Database (http://ted.bti.cornell.edu/). Probe Match tool in NetAffx Analysis Center (http://www.affymetrix.com) was used to obtain probe sequences. The average value was considered for RING genes that had more than one probe set. Total RING genes identified in the microarray data are listed in Additional File 6.

## Figures and Tables

**Table 1:** The types and properties of RING domains in *S. lycopersicum*.

RING Domain		Consensus Sequence														
Type	No.	ml1		ml2		ml3		ml4		ml5		ml6		ml7		ml8
RING-H_2_	248	C	X_2_	C	X_11-33_	C	X_1_	H	X_2,3_	H	X_2_	C	X_7-64_	C	X_2_	C
RING-HCa	142	C	X_2_	C	X_10-33_	C	X_1-3_	H	X_2,3_	C	X_2_	C	X_6-30_	C	X_2_	C
RING-HCb	21	C	X_2_	C	X_11-18_	C	X_1,2_	H	X_2_	C	X_2_	C	X_7-19_	C	X_3,4_	C
RING-v	40	C	X_2_	C	X_8-29_	C	X_1-4_	C	X_4-7,9_	H	X_2_	C	X_9-36_	C	X_2_	C
RING-C2	20	C	X_2_	C	X_7-16_	C	X_1-3_	C	X_4_	C	X_2_	C	X_10-16_	C	X_2_	C
RING-S/T	2	C	X_2_	C/S	X_11_,_14_	C	X_1_	H	X_2,3_	C	X_2_	S	X_10_,_14_	C	X_2_	C
RING-G	1	C	X_2_	C	X_16_	C	X_1_	H	X_2_	G	X_2_	C	X_13_	C	X_2_	C

The eight conserved metal–ligand (ml) positions in canonical and modified RING domains are colored blue. Spacing between the two residues in each Zn^2+^-coordinating amino acid pair is highlighted in red. X(n) indicates the number of amino acids between the conserved metal–ligands.

## Supplementary Materials

Supplementary materials can be found at https://www.mdpi.com/1422-0067/20/19/4864/s1.
