# Supplementary figures and images for: Genome-Wide Identification, Evolution, and Expression Analysis of RING Finger Gene Family in Solanum lycopersicum
Source: Int J Mol Sci. 2019 Sep 30;20(19):4864. doi: 10.3390/ijms20194864 (PMC6801689; doi:10.3390/ijms20194864)

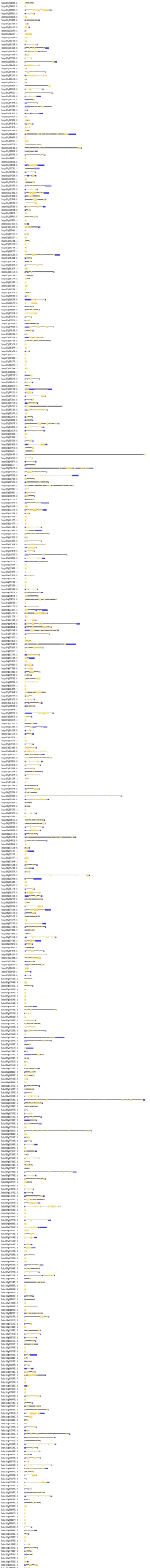

Solyc12g040390.2.1  
Solyc12g040760.1.1  
Solyc12g042790.1.1  
Solyc12g049330.2.1  
Solyc12g055710.1.1

Supplement: Supplementary file 1 [file ijms-20-04864-s001.zip › Figure S1.pdf]
